# The transcript expression levels of *HNRNPM*, *HNRNPA0* and *AKAP17A* splicing factors may be predictively associated with ageing phenotypes in human peripheral blood

**DOI:** 10.1007/s10522-019-09819-0

**Published:** 2019-07-10

**Authors:** Benjamin P. Lee, Luke C. Pilling, Stefania Bandinelli, Luigi Ferrucci, David Melzer, Lorna W. Harries

**Affiliations:** 1 Institute of Biomedical and Clinical Sciences, University of Exeter College of Medicine and Health, RILD Building, RD&E NHSFT Campus, Barrack Rd, Exeter, EX2 5DW UK; 2 Epidemiology and Public Health, University of Exeter College of Medicine and Health, RILD Building, RD&E NHSFT Campus, Barrack Rd, Exeter, EX2 5DW UK; 3 Geriatric Unit, USL Toscana Centro, 50122 Florence, Italy; 4 National Institute on Aging, Clinical Research Branch, Harbor Hospital, Baltimore, MD 21225 USA

**Keywords:** Splicing factors, Cognitive decline, Biomarkers

## Abstract

Dysregulation of splicing factor expression is emerging as a driver of human ageing; levels of transcripts encoding splicing regulators have previously been implicated in ageing and cellular senescence both in vitro and in vivo. We measured the expression levels of an a priori panel of 20 age- or senescence-associated splicing factors by qRT-PCR in peripheral blood samples from the InCHIANTI Study of Aging, and assessed longitudinal relationships with human ageing phenotypes (cognitive decline and physical ability) using multivariate linear regression. *AKAP17A, HNRNPA0* and *HNRNPM* transcript levels were all predictively associated with severe decline in MMSE score (*p* = 0.007, 0.001 and 0.008 respectively). Further analyses also found expression of these genes was associated with a performance decline in two other cognitive measures; the Trail Making Test and the Purdue Pegboard Test. *AKAP17A* was nominally associated with a decline in mean hand-grip strength (*p* = 0.023), and further analyses found nominal associations with two other physical ability measures; the Epidemiologic Studies of the Elderly-Short Physical Performance Battery and calculated speed (m/s) during a timed 400 m fast walking test. These data add weight to the hypothesis that splicing dyregulation may contribute to the development of some ageing phenotypes in the human population.

**Electronic supplementary material:**

The online version of this article (10.1007/s10522-019-09819-0) contains supplementary material, which is available to authorized users.

## Introduction

There is an intimate relationship between stress responses and successful ageing (Kourtis and Tavernarakis 2011) yet the ability to respond appropriately to stressful environments and to maintain systemic homeostasis declines with age in multiple species (Kirkland et al. 2016; Schorr et al. 2018; Varadhan et al. 2008). Cellular responses to external and internal stressors are mediated at the level of genomic plasticity, in particular at the level of the transcriptome. Several mechanisms are known to play a part in the diversity of response, including transcriptional regulation at the level of polymerase activity (Chen et al. 2018), post-transcriptional regulation (Harvey et al. 2017), epigenetics (Guillaumet-Adkins et al. 2017) and genomic landscape (Winick-Ng and Rylett 2018). Alternative splicing comprises a key part of the homeostatic response to stress (Disher and Skandalis 2007; Martinez and Lynch 2013; Mastrangelo et al. 2012), and dysregulation of this process is now emerging as a new and important driver of cellular ageing (Harries et al. 2011; Holly et al. 2013; Latorre and Harries 2017). Over 95% of genes are capable of producing more than one mRNA product under different conditions and alternatively-expressed mRNAs can have profoundly different temporal or spatial expression patterns, or demonstrate major differences in functionality (Celotto and Graveley 2001; Grumont and Gerondakis 1994; Pan et al. 2008).

Alternative splicing decisions are made by a series of trans-acting splicing regulatory proteins termed splicing factors. These are the Serine Arginine-rich (SR) family of splicing factors which usually, but not exclusively, promote splice site usage, and the heterogeneous nuclear ribonucleoprotein (HNRNP) family of splicing factors which are usually, but not exclusively, associated with inhibition of splice site usage (Cartegni et al. 2002). SR proteins and HNRNPs bind to exon/intron splicing enhancer (ESE/ISE) or silencer (ESS/ISS) elements in the vicinity of the splice sites and the balance of activators and inhibitors at any given splice site regulates splice site usage (Smith and Valcarcel 2000). The expression levels of splicing regulators is known to be associated with ageing; of seven gene ontology pathways robustly associated with age in a large cross-sectional population study of human ageing, six were directly involved in mRNA splicing processes (Harries et al. 2011). Splicing factor expression is also associated with lifespan in mice and humans (Lee et al. 2016).

We have previously observed disruption of splicing factor expression in human senescent cells (Holly et al. 2013; Latorre et al. 2018b), and demonstrated that experimental manipulation of splicing factor expression is capable of inducing rescue from the senescent cell phenotype (Latorre et al. 2017, 2018a, c). Although we have demonstrated epidemiological links with ageing itself, and reversal of cellular senescence in vitro, evidence that the phenomena we observe in vitro is linked with downstream ageing phenotypes is lacking. In this study, we addressed this question by measurement of the expression of an a priori panel of age- and senescence-related splicing factor genes in human peripheral blood mRNA from the InCHIANTI study of Aging. We used samples from two follow-up visits (FU3; 2007–2010 and FU4; 2012–2014) of the InCHIANTI study of Aging, and related their expression to changes in recorded measures of two important human ageing phenotypes; cognitive and physical function. We initially used the Mini Mental State Exam (MMSE) score and mean hand-grip strength to identify putative associations between splicing factor expression and changes in cognitive or physical ability respectively. We then assessed expression of these transcripts against other cognitive and physical measures available in the dataset. In each case, a set of sub-analyses were also performed to test the robustness of the findings.

We found that the expression of three splicing factor genes, *HNRNPM*, *HNRNPA0* and *AKAP17A* may be predictive for change in in this population; all three genes were associated with cognitive decline as measured by the Mini-Mental State Examination (MMSE), Trail-Making Tests part A and B (TMT A/B), and the Purdue Pegboard Test (PPT). *AKAP17A* was also associated with a decline in physical ability as measured by hand-grip strength, the Epidemiologic Studies of the Elderly-Short Physical Performance Battery (EPESE-SPPB) and calculated speed during a timed 400 m fast walking test. Our data suggest that age-associated dysregulation of splicing factor expression in ageing humans may contribute to the development of downstream ageing outcomes.

## Methods

### InCHIANTI cohort and selection of participants

The InCHIANTI study of Aging is a population study of ageing (Ferrucci et al. 2000). Participants undertook detailed assessment of health and lifestyle parameters at baseline, and again at three subsequent follow-ups (FU2; 2004–2006, FU3; 2007–2010 and FU4; 2012–2014). The present study used participants from the third and fourth follow-up visits (FU3 and FU4). RNA samples and clinical/phenotypic data were already available for 698 participants at FU3. The collection of the FU4 samples and data comprise part of this study. During the FU4 interviews in 2012/13, blood and clinical/phenotypic data were collected from 455 study participants. These data were cross-checked against RNA samples and clinical/phenotypic data already held from FU3, to ensure that sample and phenotypic data was available from both collections. 393 individuals fitted these criteria, of which nine died shortly after the FU4 visit and so were excluded from the analysis. From the remaining 384 eligible samples, 300 were randomly selected from the cohort to be analysed for expression of splicing factor genes. Anthropometric parameters and blood cell subtypes in FU4 were measured as previously (Ferrucci et al. 2000).

### Splicing factor candidate genes for analysis

An a priori list of splicing factor candidate genes were chosen based on associations we had documented with human ageing in multiple populations and in senescent primary human cell lines in our previous work (Harries et al. 2011; Holly et al. 2013; Latorre et al. 2017). We have also found associations of components of this gene set with lifespan in both mice and humans (Lee et al. 2016). The list of genes included the positive regulatory splicing factors *AKAP17A*, *SRSF1*, *SRSF2*, *SRSF3*, *SRSF6*, *SRSF7*, *PNISR* and *TRA2B*, the negative regulatory splicing inhibitors *HNRNPA0*, *HNRNPA1*, *HNRNPA2B1*, *HNRNPD*, *HNRNPH3*, *HNRNPK*, *HNRNPM*, *HNRNPUL2* and the *IMP3*, *LSM14A*, *LSM2* and *SF3B1* core components of the spliceosome. Expression assays were obtained in custom TaqMan^®^ low-density array (TLDA) format (ThermoFisher, Waltham, MA, USA). Assay Identifiers are given in Supplementary Table S1.

### RNA collection and extraction

2.5 ml of peripheral blood was collected from each participant into PAXgene Blood RNA Tubes (IVD) (PreAnalytiX GmbH, Hombrechtikon, Switzerland). Blood tubes were then treated according to the manufacturer’s instructions and subsequently cold-chain shipped to the UK. RNA extractions were then carried out using the PAXgene Blood mRNA Kit (Qiagen, Hilden, Germany), according to manufacturer’s instructions. Samples were assessed for RNA quality and quantity by Nanodrop spectrophotometry (NanoDrop, Wilmington, DE, USA).

### Reverse transcription and quantitative RT-PCR

100 ng of total RNA was reverse transcribed using SuperScript^®^ VILO™ cDNA Synthesis Kit (ThermoFisher, Waltham, MA, USA) in 20 μl reactions, according to the manufacturer’s instructions. 20 μl cDNA (reverse transcribed as indicated above) was added to 50 μl TaqMan^®^ Universal Master Mix II, no UNG (ThermoFisher, Waltham, MA, USA) and 30 μl RNase-free dH_2_O, then loaded onto TaqMan^®^ Low-Density Array 384-Well Microfluidic cards. 100μL reaction solution was dispensed into each TLDA card chamber and the card centrifuged twice for 1 min at 216×*g* to ensure distribution of solution to each well. The expression of transcripts in each sample was measured in duplicate replicates. Cards were run on the 7900HT Fast Real-Time PCR System (ThermoFisher, Waltham, MA, USA). Amplification conditions were as follows: a single cycle of 50 °C for 2 min, a single cycle of 94.5 °C for 10 min followed by 40 cycles of 97 °C for 30 s and 59.7 °C for 1 min.

### Data preparation

SDS files were uploaded to the ThermoFisher Cloud (ThermoFisher, Waltham, MA, USA) and analysed using the Relative Quantification qPCR App encompassed within the software (https://www.thermofisher.com/uk/en/home/cloud.html). This platform was used to manually set Baseline and Threshold for each assay (see Supplementary Table S1 for values) and to ensure there were no apparent outliers before further analysis. One sample was excluded at this stage as expression data was missing for all genes measured. Output was imported into Excel (Microsoft, Redmond, WA, USA) and the C_T_ values used for analysis using the comparative C_T_ method. The most stable genes for use as endogenous controls were determined from the raw data using the RefFinder webtool (Xie et al. 2012), which returned the geometric mean value across all genes measured as the most stable control, and thus the most appropriate for the ΔC_T_ normalisation step. Expression was then calculated relative to the median expression for each individual transcript. Data were log transformed to ensure normal distribution. Outlier detection was performed in SPSS (IBM, Armonk, NY, USA). Univariate outliers were identified using standardised z-scores, with any individual measures for each gene falling outside the cut-off (set at three standard deviations from the mean) being discarded. Multivariate outliers were identified using a regression model with Mahalanobis distance as an output, followed by comparison of the calculated Mahalanobis distances with the critical χ^2^ value for the dataset (Rasmussen 1988). One sample for which the Mahalanobis distance exceeded the critical χ^2^ was discarded, leaving a total of n = 298 samples to take forward for statistical testing. The characteristics of this final subset of participants are summarised in Table 1.

**Table 1:** Participant details

(A)				
	Follow-up 3		Follow-up 4	
	n	%	n	%
Participants	298	100	298	100
Age (years)				
30–39	21	7.05	11	3.69
40–49	31	10.40	28	9.40
50–59	31	10.40	28	9.40
60–69	35	11.74	34	11.41
70–79	111	37.25	38	12.75
80–89	67	22.48	142	47.65
90–100	2	0.67	17	5.70
Gender				
Male	136	45.64	136	45.64
Female	162	54.36	162	54.36
Pack years smoked (lifetime)				
None	159	53.36	No	Data
< 20	79	26.51	No	Data
20–39	44	14.76	No	Data
40 +	16	5.37	No	Data
Site				
Greve	140	46.98	140	46.98
Bagno a Ripoli	158	53.02	158	53.02
Education level attained				
Nothing	28	9.4	24	8.05
Elementary	121	40.6	125	41.95
Secondary	51	17.11	59	19.80
High school	46	15.44	58	19.46
Professional school	33	11.07	11	3.69
University or equivalent	19	6.38	21	7.05

(B)								
	Follow-up 3				Follow-up 4			
	Mean	Std. dev.	Min	Max	Mean	Std. dev.	Min	Max
Age (years)	67.69	15.68	30.00	94.00	72.92	15.68	35.00	100.00
BMI	26.97	4.27	15.01	42.99	26.86	4.42	13.39	41.19
White blood cell count (n, K/μl)	6.39	1.60	2.10	13.00	6.16	1.72	2.30	16.59
Neutrophils (%)	56.61	8.61	26.20	81.20	56.50	9.23	22.20	88.40
Lymphocytes (%)	31.56	8.04	9.80	59.90	32.47	8.70	8.30	63.30
Monocytes (%)	8.08	2.22	3.70	21.30	7.36	2.28	1.60	24.40
Eosinophils (%)	3.19	2.20	0.00	21.50	3.21	2.02	0.00	13.00
Basophils (%)	0.55	0.20	0.10	1.30	0.47	0.29	0.00	2.10

(C)									
	n	Follow-up 3				Follow-up 4			
		Mean	Std. dev.	Min	Max	Mean	Std. dev.	Min	Max
Corrected MMSE score	296	27.22	3.18	14.00	30.00	25.71	5.11	0.00	30.00
TMT-A (mins)	268	0.91	0.62	0.23	5.00	1.13	0.83	0.25	5.00
TMT-B (mins)	179	1.56	1.02	0.47	5.00	1.91	1.19	0.52	5.00
PPT (total pegs placed)	257	64.77	13.38	26	98	61.60	16.45	17	103
Mean hand-grip strength (Kg)	285	29.67	12.28	10.00	70.75	28.11	12.14	5.00	65.50
EPESE-SPPB (composite score)	276	11.10	1.59	0	12	10.09	2.95	0	12
400 m fast walk speed (m/s)	206	1.41	0.25	0.73	2.18	1.26	0.29	0.47	2.03

Characteristics of the InCHIANTI participants used in the present study. Panel A shows summary of non-clinical details, panel B shows summary results of clinical/laboratory tests and panel C shows summary results of phenotypic measures used for analysis

### Phenotypic outcomes for analysis

The current study analysed the associations of splicing factor gene expression at FU3 with the following cognitive phenotypic outcomes; MMSE score, Trail-Making-Test (TMT A&B) and Purdue Pegboard Test (as measures of cognitive function), along with hand-grip strength, EPESE-SPPB composite score and calculated speed during a 400 m fast walk (as measures of physical ability).

MMSE score was measured at both FU3 and FU4 using the standard test, after which the data was corrected to adjust for incomplete tests. This was calculated using the score attained as a proportion of the maximum possible points for the parts of the test that were completed. Decline in MMSE score was calculated by subtracting the score at FU4 from the score at FU3. Time taken to complete the Trail-Making-Tests part A and B were measured in seconds at both FU3 and FU4 using the standard tests. Decline in performance on the tests was calculated by subtracting the score at FU4 from the score at FU3. Decline in seconds was then converted to a decline in fractions of a minute prior to analysis. The Purdue Pegboard Test was administered as standard (although data for the assembly portion of the test was not available), and scores for number of pegs placed in the board for right-hand, left-hand and both-hands were summed to give a total number of pegs placed during the test. Decline in performance on the test was calculated by subtracting the total number of pegs placed at FU4 from the total number of pegs placed at FU3.

Hand-grip strength was measured in kilograms at both FU3 and FU4 using a dynamometer, with two separate measurements taken for each hand. Mean hand-grip strength was used for the analyses in this study, and was calculated as the mean of all 4 hand-grip strength measurements across both hands. Decline in mean hand-grip strength was calculated by subtracting the measurement at FU4 from the measurement at FU3. The EPESE-SPPB was administered and scored as described elsewhere (Guralnik et al. 1994), and a composite score generated from the sub-scores of the three activities performed: repeated chair-stand, standing balance and 4 m normal pace walk. Decline in performance was calculated by subtracting the composite score at FU4 from the composite score at FU3. The 400 m fast walk was performed by completing 20 laps of a 20 m circuit with a maximum of two stops if the subject required. Speed in m/s was calculated over the entire distance (any individuals who did not complete the test were excluded from further analysis), and decline in performance was calculated by subtracting the speed at FU4 from the speed at FU3.

### Sub-analyses for robustness testing

For all phenotypes, any associations found in the full cohort were then tested for robustness through four sub-analyses on different subsets of the data.

First, individuals with the lowest initial scores (at FU3) were excluded from the analysis, to avoid confounding due to the inclusion of participants already on a trajectory to decline. For MMSE score, the cut-off was set at ≥28, as a score above 28 is clearly indicative of a lack of cognitive impairment. In the case of mean hand-grip strength, the cut-offs used were those previously reported as a consensus definition of sarcopenia by The European Working Group on Sarcopenia in Older People (EWGSOP) (Cruz-Jentoft et al. 2010), i.e. < 20 kg for females and < 30 kg for males. For all the other phenotypes analysed, the dataset at FU3 was divided into quintiles, with the lowest scoring quintile being excluded from this sub-analysis.

Second, an analysis was carried out using only the eldest participants aged ≥ 70 years at FU3 to exclude any potential confounding effects from younger participants. Declining cognitive and physical ability are predominantly features of ageing, and measures such as mean hand-grip strength and MMSE are likely to perform poorly in measuring change of function in young, non-compromised individuals.

Third, some individuals measured showed an apparent improvement in performance over time between FU3 and FU4, which may reflect a degree of measurement error. To test this, we first calculated an allowed error of 5% (as a fraction of the total range of change measured), then removed any individuals with scores showing an increase greater than the allowed error between the follow-ups, for each phenotype.

Finally, participants were categorised into mild or severe decline classes, to assess whether the associations seen were specific to either group of individuals. Decline in MMSE score was categorised for sub-analysis as follows; ‘No decline’ (score change of − 1 to + 7, based on a 5% allowable error calculated as described above), ‘Mild decline’ (− 2 to − 8), and ‘Severe decline’ (− 9 to − 22). While opinion differs as to what amounts to a significant rate of change in MMSE during cognitive decline, we chose to classify a severe decline as a drop in MMSE score of > 3 per annum on average, based on information from several previous studies (Clark et al. 1999; Hensel et al. 2007; McCarten et al. 2004). Analysis of categorised MMSE decline was carried out using the ‘No decline’ class as the comparator.

Decline in mean hand-grip strength was categorised as follows; quintiles of change in mean hand-grip were calculated separately for males and females, after which the respective 20% of males and females displaying the largest decline in mean hand-grip strength were together designated as the ‘Severe decline’ class (mean hand-grip change of − 3.75 kg to − 22 kg). The remainder of the cohort was divided into ‘No decline’ (− 1 kg to +15.5 kg, based on a 5% allowable error calculated as described above) and ‘Mild decline’ (− 1.25 kg to − 6 kg) categories. Analysis of categorised mean hand-grip strength decline was carried out using the ‘No decline’ class as the comparator.

For all other measures used for analysis, categorisation was carried out by dividing using the cohort into quintiles based on the change in score, and the quintile with the greatest decline in performance designated as the ‘Severe decline’ class. The remainder of the cohort was divided into ‘No decline’ and ‘Mild decline’ classes using the same method as described above for MMSE and mean hand-grip strength. Analysis of categorised variables was carried out using the ‘No decline’ class as the comparator in all cases.

### Statistical analysis

Associations of gene expression with cognitive and physical phenotype measures were assessed using multivariate linear regression models. All models were adjusted for age, sex, BMI, smoking (lifetime pack-years), highest education level attained, study site, TLDA batch and cell counts (neutrophils, lymphocytes, monocytes, eosinophils and overall white blood cell count). Regressions were carried out in STATA SE v15.1 (StataCorp, College Station, TX, USA). Pearson correlation tests were carried out in SPSS (IBM, Armonk, NY, USA) to assess relationships between splicing factor expression levels, phenotypic measures and established biomarkers of ageing.

## Results

### AKAP17A, HNRNPA0 and HNRNPM transcript levels are associated with change in MMSE score

**Fig. 1:**
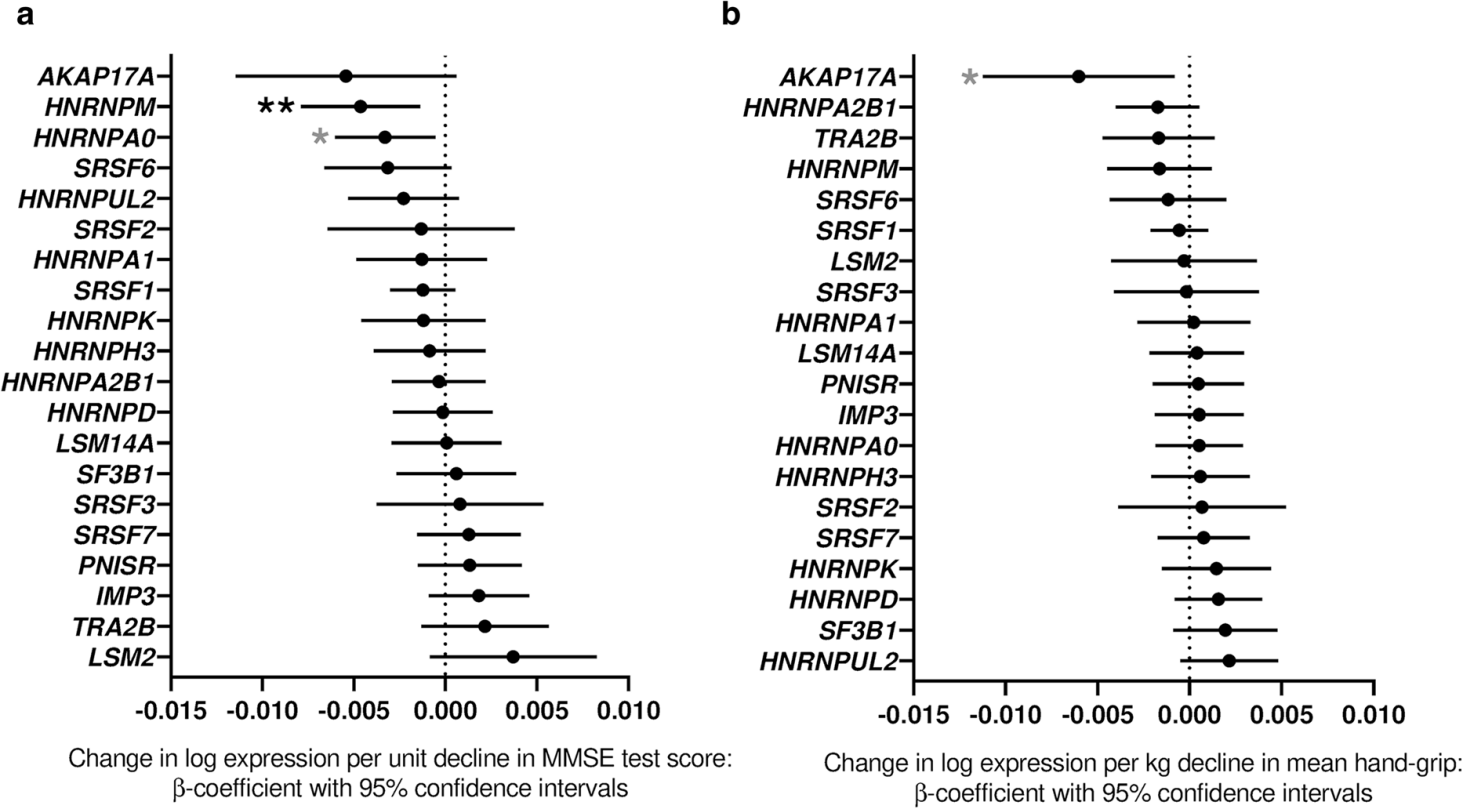
Associations of splicing factor expression with MMSE score. Forest plots showing splicing factor expression in relation to **a** decline in corrected MMSE scores and **b** mean hand-grip strength in the InCHIANTI human ageing cohort. Individual splicing factors are indicated on the *y*-*axis* while β-coefficients of change in log expression per unit change in measurements are given on the *x*-*axis*. Positive values denote an increase in expression with larger decline in score while negative values denote a decrease in expression with larger decline in score. Error bars denote 95% confidence intervals, significance is shown using stars as follows: *p < 0.05, **p < 0.01. Stars indicated in black denote associations which meet multiple testing thresholds, while those in grey represent nominal associations

MMSE is a commonly used measure of cognitive decline (Clark et al. 1999; Hensel et al. 2007; McCarten et al. 2004). *HNRNPM* expression showed a significant association with decline in MMSE score in the entire cohort (β-coefficient − 0.005, *p* = 0.006), with *HNRNPA0* also showing a nominal association (β-coefficient − 0.003, *p* = 0.019). In both cases individuals with lower expression levels at the early time-point (FU3) had subsequently experienced a greater drop in MMSE score (Fig. 1a, Supplementary Table S2). The remaining splicing factor genes did not demonstrate associations between MMSE and expression.

To test the robustness of our findings we carried out four sub-analyses. Similar sub-analyses were also used on all other associations found in the present study and full details can be found in the Methods. In brief, regression models were repeated on the following subsets of data: firstly we removed individuals with low starting scores, secondly only individuals over 70 years of age were included, thirdly any individuals showing an increase in performance over time were excluded, and finally participants were categorised into ‘mild’ or ‘severe’ decline classes.

**Fig. 2:**
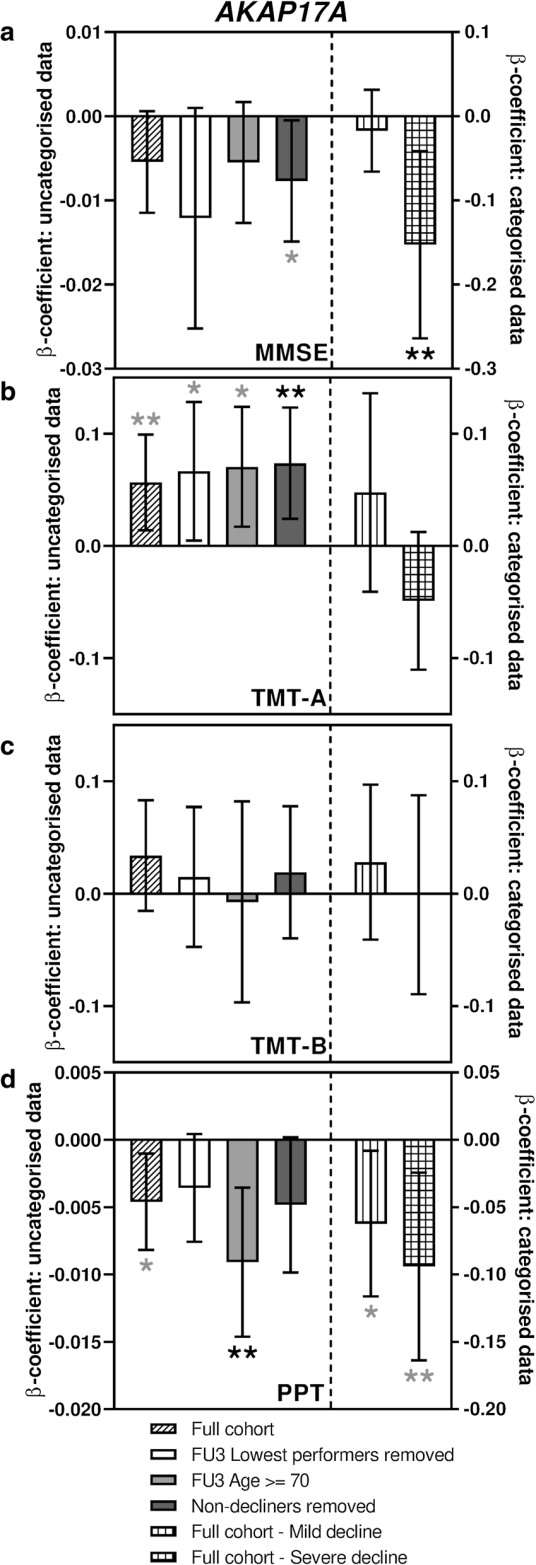
Sub-analyses of *AKAP17A* associations with measures of cognitive function. Bar charts showing the associations between expression of *AKAP17A* and change in performance in tests of cognitive function. **a** shows associations with MMSE score, **b** shows associations with TMT-A, **c** shows associations with TMT-B and **d** shows associations with PPT. Different sub-analyses are plotted separately as indicated in the figure legend. Error bars denote 95% confidence intervals, significance is shown using stars as follows: *p < 0.05, **p < 0.01. Stars indicated in black denote associations which meet multiple testing thresholds, while those in grey represent nominal associations

**Fig. 3:**
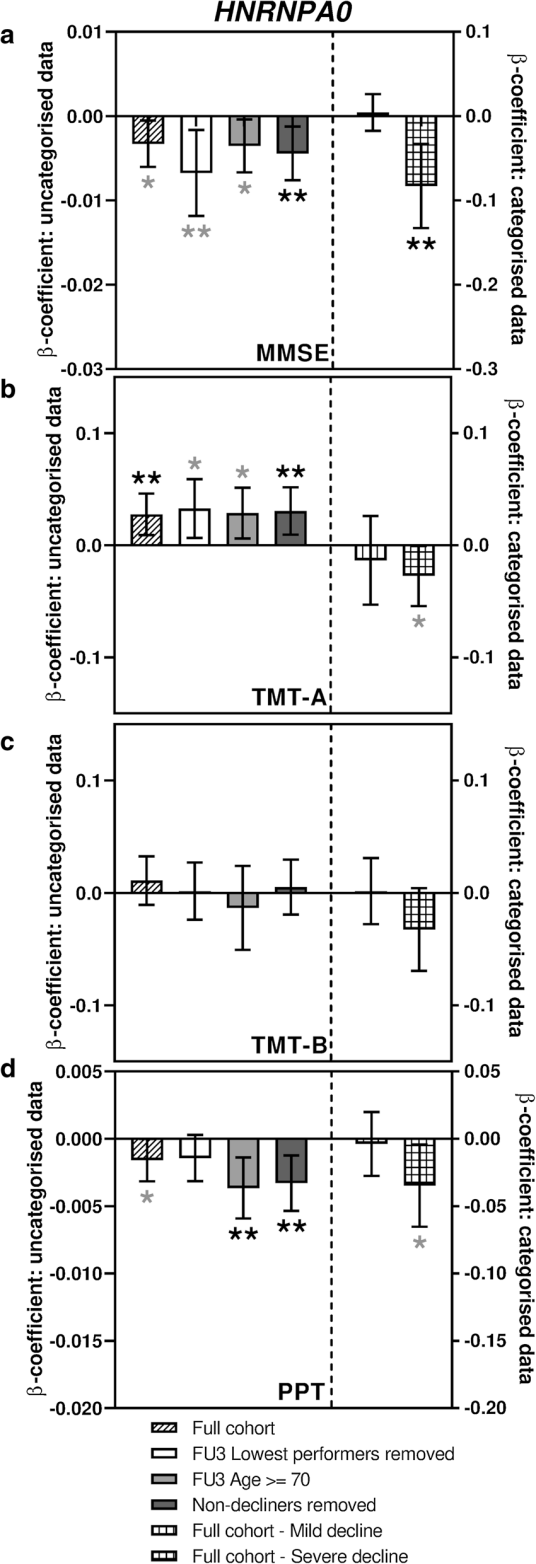
Sub-analyses of *HNRNPA0* associations with measures of cognitive function. Bar charts showing the associations between expression of *HNRNPA0* and change in performance in tests of cognitive function. **a** shows associations with MMSE score, **b** shows associations with TMT-A, **c** shows associations with TMT-B and **d** shows associations with PPT. Different sub-analyses are plotted separately as indicated in the figure legend. Error bars denote 95% confidence intervals, significance is shown using stars as follows: *p < 0.05, **p < 0.01. Stars indicated in black denote associations which meet multiple testing thresholds, while those in grey represent nominal associations

**Fig. 4:**
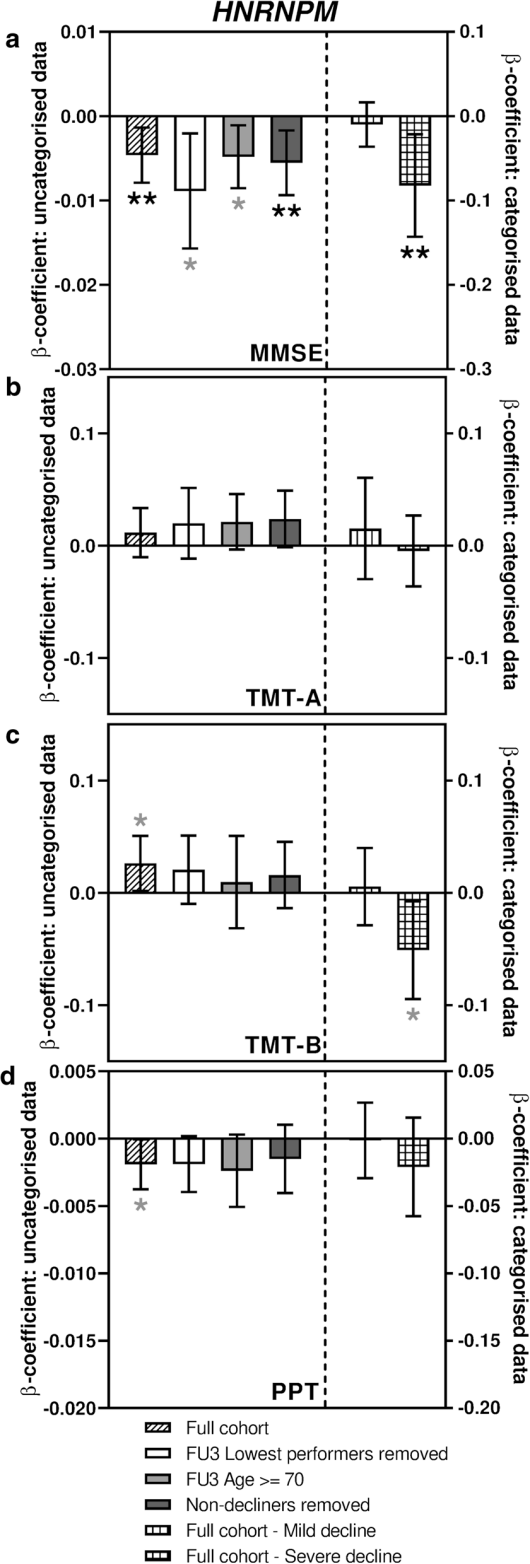
Sub-analyses of *HNRNPM* associations with measures of cognitive function. Bar charts showing the associations between expression of *HNRNPM* and change in performance in tests of cognitive function. **a** shows associations with MMSE score, **b** shows associations with TMT-A, **c** shows associations with TMT-B and **d** shows associations with PPT. Different sub-analyses are plotted separately as indicated in the figure legend. Error bars denote 95% confidence intervals, significance is shown using stars as follows: *p < 0.05, **p < 0.01. Stars indicated in black denote associations which meet multiple testing thresholds, while those in grey represent nominal associations

Although *AKAP17A* showed only a trend with MMSE decline in the initial analysis, the observation that it had the largest β-coefficient, coupled with a suggestive *p* value of 0.077 merited its inclusion in these further analyses. As can be seen in Figs. 2a, 3a and 4a (Supplementary Table S3), both *HNRNPA0* and *HNRNPM* remained at least nominally associated with decline in MMSE score across all sub-analyses, and significantly associated with severe decline in the categorised analysis. *AKAP17A* on the other hand was only nominally associated with decline in MMSE in the sub-analysis excluding the individuals displaying an apparent improvement over time, but in the categorised analysis a significant association was also seen between *AKAP17A* expression and severe decline.

To assess whether these findings represent independent effects or could be driven by co-ordinate expression of the three genes, we carried out correlation analysis. Correlations between the three genes in question were only moderate (R values: *HNRNPA0* & *HNRNPM*: 0.282, *HNRNPA0* & *AKAP17A*: 0.239, *HNRNPM* & *AKAP17A*: 0.440 (Supplementary Table S4).

### Expression of HNRNPA0, HNRNPM and AKAP17A transcripts are also associated with two other measures of cognitive ability

The Trail Making Test (TMT) is another widely used test for cognitive assessment which addresses visual scanning, graphomotor speed and executive function (Llinas-Regla et al. 2017). Figures 2b, c, 3b, c and 4b, c (Supplementary table S5) show *AKAP17A* and *HNRNPA0* transcript levels were at least nominally associated with increased time to complete TMT-A, both in the full cohort (β-coefficients 0.057 and 0.028, *p* = 0.009 and 0.004 for *AKAP17A* and *HNRNPA0* respectively) and in all sub-analyses with the exception of the categorised analysis for *AKAP17A*. *HNRNPM* expression was nominally associated with increased time to complete TMT-B, but only in the full cohort (β-coefficient 0.026, *p* = 0.036) and categorised analyses. In all cases, lower expression levels were associated with an increase in the time taken to complete the test (i.e. a decline in performance).

The Purdue Pegboard Test (PPT) was originally developed as a tool to evaluate fine manual dexterity but has since been used for assessments of cognitive function (Brown et al. 1993; Zakzanis et al. 1998). As shown in Figs. 2d, 3d and 4d (Supplementary table S5), all three transcripts were nominally associated with a performance decline in the full cohort (β-coefficients − 0.005, − 0.002 and − 0.002, *p* = 0.012, 0.047 and 0.044 for *AKAP17A*, *HNRNPA0* and *HNRNPM* respectively). While *AKAP17A* and *HNRNPA0* transcript levels were found to be significantly associated with performance decline in some of the sub-analyses, *HNRNPM* showed no such further associations.

Pearson correlations were also carried out to assess relationships between the aspects of cognition being measured by MMSE, TMT and PPT. Correlations between the measures were relatively weak (R values range from − 0.381 to 0.322, see Supplementary Table S6a).

### Expression of AKAP17A transcript is associated with mean hand-grip strength

**Fig. 5:**
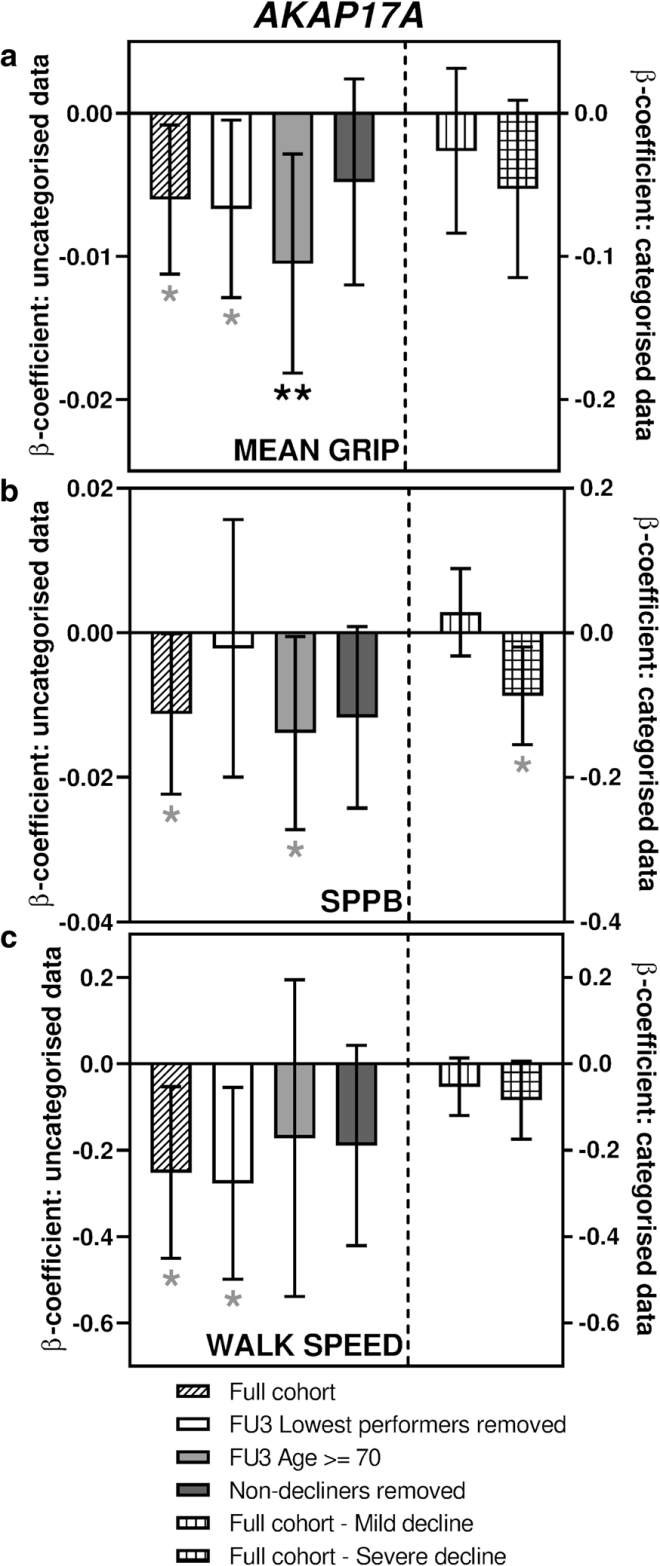
Sub-analyses of *AKAP17A* associations with measures of physical ability. Bar charts showing the associations between expression of *AKAP17A* and change in performance in tests of physical ability. **a** shows associations with mean hand-grip strength, **b** shows associations with the EPESE-SPPB composite score and **c** shows associations with calculated speed (m/s) during a 400 m fast walking test. Different sub-analyses are plotted separately as indicated in the figure legend. Error bars denote 95% confidence intervals, significance is shown using stars as follows: *p < 0.05, **p < 0.01. Stars indicated in black denote associations which meet multiple testing thresholds, while those in grey represent nominal associations

Hand-grip strength, a measure of muscle weakness, is a useful indicator of physical functioning and health-related quality of life in the elderly (Bohannon 2015). Of the transcripts tested, only *AKAP17A* transcript levels were nominally associated with decline in hand-grip strength between FU3 and FU4 (β-coefficient − 0.006, *p* = 0.023) (Fig. 1b, Supplementary Table S7). As shown in Fig. 5a (Supplementary Table S8), robustness testing of this finding revealed one nominal and one significant association in the sub-analyses, and no associations with the categorised data.

### Expression of AKAP17A transcript is also associated with two other measures of physical ability

The Epidemiologic Studies of the Elderly-Short Physical Performance Battery (EPESE-SPPB) is a validated measure of lower body function and is predictive of several important health outcomes, including mortality (Pavasini et al. 2016). *AKAP17A* expression was found to be nominally associated with decline in the EPESE-SPPB composite score (β-coefficient − 0.011, *p* = 0.048), and as can be seen in Fig. 5b (Supplementary Table S9), subsequent testing showed it also to be nominally associated in two of the sub-analyses.

Another measure of physical ability that was available in the data set was the calculated speed (m/s) during a timed 400 m fast walking test. Once again, *AKAP17A* expression was found to be nominally associated with decline in walking speed (β-coefficient − 0.252, *p* = 0.013). Figure 5c (Supplementary Table S9) shows that this nominal association only held true in one sub-analysis.

Finally, Pearson correlations were carried out to assess relationships between the aspects of physical ability being measured. Again, correlations between the measures were relatively weak (R values range from 0.217 to 0.357, see Supplementary Table S6b).

### *HNRNPA0*, *HNRNPM* and *AKAP17A* transcripts show correlations with known biomarkers of ageing

To test whether these transcripts might share expression patterns with levels of recognised biomarkers of ageing (Xia et al. 2017), where such measures were available in our dataset, we carried out Pearson correlations between transcript expression levels and measurements of interleukin-6 (IL-6), albumin and total erythrocyte numbers. Significant correlations were found between: *AKAP17A* expression and IL-6 levels (R value = 0.144, p = 0.013); *HNRNPA0* expression and both IL-6 and erythrocyte number (R values = − 0.200 and 0.115, p = 0.001 and 0.048 respectively); *HNRNPM* expression and albumin levels (R value = − 0.137, p = 0.018).

## Discussion

The human genome is equipped with mechanisms to generate transcriptomic diversity from a relatively small DNA complement (Nilsen and Graveley 2010). This transcriptomic diversity underpins our ability to respond appropriately to internal and external environmental challenges, while the failure of such cellular stress responses contributes to ageing itself and to age-related diseases (Kourtis and Tavernarakis 2011). Alternative splicing is one of the major mechanisms for generation of such diversity (Kelemen et al. 2013), and is regulated by the combinatorial binding of a set of trans-acting activating and inhibitory proteins termed splicing factors to *cis*- sequence control elements (Smith and Valcarcel 2000). Dysregulation of splicing factor expression occurs with human ageing at the epidemiological and cellular levels (Harries et al. 2011; Holly et al. 2013), and is also associated with longevity in animal models (Lee et al. 2016). These changes are drivers of cellular ageing, since restoration of splicing factor levels is able to reverse multiple senescence phenotypes in aged human cells in vitro (Latorre et al. 2017, 2018a, c). In the work described here, we provide evidence that changes in splicing factor expression are not only present in in vitro data, but may also contribute to the development of downstream ageing phenotypes in older people. We report predictive associations between the transcript expression levels of three splicing factor genes, *HNRNPM*, *HNRNPA0* and *AKAP17A* with several ageing phenotypes in a human population study, the InCHIANTI study of Aging (Ferrucci et al. 2000).

*HNRNPM*, *HNRNPA0* and *AKAP17A* transcript levels were predictively associated with a decline in MMSE score as well as a decline in performance on the Trail-Making Test parts A & B and the Purdue Pegboard Test. Associations between transcript expression of all three genes and ageing phenotypes were strongest in the individuals with severe cognitive decline as measured by MMSE. It is possible that these three splicing factors are not independently associated with the traits in question, but correlations between them are only moderate. *HNRNPM* and *HNRNPA0* encode splicing inhibitor proteins that have roles in determining the splicing patterns of several genes with relevance to brain physiology. TDP-43 is one of the major proteins involved with Amyotrophic lateral sclerosis (ALS) and Frontotemporal Dementia (FTD) in humans (Orr 2011). HNRNPM and HNRNPA0 are both known to interact with TDP-43 (Couthouis et al. 2011), and mutations in other HNRNPs have been described in patients with ALS (Calini et al. 2013). In *Drosophila* species, depletion of the fly homologue of *HNRNPM* (*Rump*) is associated with loss of neuronal dendritic terminal branches; a phenotype that could be partially restored by the addition of a Rump transgene (Xu et al. 2013). HNRNPM has also been demonstrated to directly regulate alternative splicing of the dopamine receptor 2 (*DRD2*) gene, whereby it inhibits the inclusion of exon 6 (Park et al. 2011). *DRD2* splice variants have previously been implicated with schizophrenia and impaired cognitive function in humans (Cohen et al. 2016; Kaalund et al. 2014). Similarly, increased protein expression of HNRNPA0 in hippocampus has been shown to be associated with memory formation and consolidation in mice (Ferreira et al. 2016). *AKAP17A*, otherwise known as *SFSR17A*, is an X-linked gene encoding a splicing regulatory factor that also has roles in targeting protein kinase A anchoring protein to splicing factor compartments (Jarnaess et al. 2009). It is a poorly characterised gene, but has been previously associated with the development of Alzheimer’s disease; sequences deriving from this gene appear twice amongst probes that best differentiate Alzheimer’s disease brain samples from controls (Lunnon et al. 2013).

*AKAP17A* was also found to be associated with decline over time in measures of physical function, although the sporadic nature of replication of this association in the sub-analyses suggests that either this finding is less robust, or the measurements themselves are more subject to variation. No functional role for *AKAP17A* has previously been reported in relation to muscle function.

Although the changes reported here represent changes in peripheral blood, similar changes to splicing regulation have been reported in senescent cell lines from other, less accessible tissues (Latorre et al. 2017, 2018b). Splicing factor expression is ubiquitous, although changes in the exact composition of the splicing factor milieu will occur from tissue to tissue. We postulate that the expression patterns of splicing factors in peripheral blood may at least partly reflect changes in less accessible tissues. We previously demonstrated that senescence-related changes in splicing factor expression in endothelial cells and cardiomyocytes are preserved in human peripheral blood, and resultant changes in the alternative splicing of the *VEGFA* gene are associated with incident and prevalent coronary artery disease (Latorre et al. 2018b). Both cognitive decline and deterioration in muscle strength also have a significant inflammatory component (Harries et al. 2012; Marottoli et al. 2017).

Our study has several strengths; the use of a longitudinal population study has allowed some assessment of causality of effect. Placed in the context of the known dysregulation of splicing factor expression in human and animal ageing, their associations with longevity in both animals and humans (Heintz et al. 2017; Lee et al. 2016) and the observation that correction of splicing factor levels is sufficient to reverse senescence phenotypes in vitro (Latorre et al. 2017) suggests that these changes may be a driver of ageing rather than an effect. The data presented here suggest that the presence of dysregulated splicing factor transcripts before the emergence of overt disease in peripheral blood may contribute to the development of age-related phenotypes such as cognitive decline. Our finding that expression of these transcripts correlate with established biomarkers of ageing is also suggestive that they may be indicative of future outcomes. Our study is not without limitations however; we have only assessed splicing factor expression at the level of the mRNA transcript for reasons of practicality. It is possible that processes such as post-transcriptional regulation of mRNA transcripts or altered protein kinetics may also contribute and would not be identified in our study. However, the observation of phenotypic changes in senescent cells in vitro strongly suggests that these changes may also occur at the protein level. Our study cohort is also relatively small in comparison to some resources. It is however exquisitely well characterised and features longitudinal waves of samples which many other studies do not. These observations require replication in an additional dataset in the future. However, we have measures from two waves of the study, which although they comprise the same people, represent completely separate sample collection, sample handling and analytical subsets.

We recognise that although an adjustment for multiple testing has been applied at the level of phenotype (i.e. accounting for six measurements) throughout this study, there remains a risk of Type I error in the results presented by using a significance level of *p *= 0.0083 as the corrected threshold. However, the genes tested represented an a priori list on the basis of known associations with age or cellular senescence (Harries et al. 2011; Holly et al. 2013), and we show here that although moderate, correlations do exist between splicing factor expression levels as well as between the measured phenotypic outcomes (Supplementary Tables S4 and S6), all of which complicate a sensible implementation of multiple testing criteria. It is also possible that the statistical power of this study may be limited given the number of samples and the observed effect sizes, leading to potential inflation of Type II error. Therefore, either Bonferroni or Benjamini–Hochberg correction for both the number of genes and phenotypes seem likely to be overly stringent for this dataset, however the issue of multiple testing persists, so without independent validation of the findings presented here, we must be conservative with any interpretation of these results.

We present here evidence to suggest that expression levels of *HNRNPM*, *HNRNPA0* and *AKAP17A* genes may be associated with cognitive decline, whilst *AKAP17A* levels may be associated with decline in physical performance in a human population. These findings suggest that the age-related splicing factor changes we have previously reported in vitro and in vivo may contribute to the development of downstream ageing phenotypes in older humans. Given validation of these findings in an independent data set, splicing factor expression could comprise a relatively non-invasive biomarker of cognitive decline or physical ability in the future, which could be assessed from samples collected from routine screening of the vulnerable people in the population.

## Electronic supplementary material

Below is the link to the electronic supplementary material.

Supplementary material 1 (DOCX 48 kb)
