# Supplementary material for: The transcript expression levels of HNRNPM, HNRNPA0 and AKAP17A splicing factors may be predictively associated with ageing phenotypes in human peripheral blood
Source: Biogerontology. 2019 Jul 10;20(5):649–63. doi: 10.1007/s10522-019-09819-0 (PMC6733819; doi:10.1007/s10522-019-09819-0)
Supplement: Supplementary file 1 — Supplementary material 1 (DOCX 48 kb) [file 10522_2019_9819_MOESM1_ESM.docx]

**Supplementary Table S1. Taqman® Low Density Array card contents.**

Splicing factor target genes, assay IDs and qPCR software settings for each transcript included on the Taqman® Low Density Array cards. Endogenous control genes used are shown in bold italics.

| Target | Assay ID | Threshold | Baseline Start | Baseline End |
| --- | --- | --- | --- | --- |
| *HNRNPA0* | Hs00246543_s1 | 0.315 | 9 | 21 |
| *HNRNPA1* | Hs01656228_s1 | 0.377 | 9 | 24 |
| *HNRNPA2B1* | Hs00242600_m1 | 0.270 | 9 | 18 |
| *HNRNPD* | Hs01086914_g1 | 0.249 | 4 | 21 |
| *HNRNPH3* | Hs01032113_g1 | 0.345 | 3 | 21 |
| *HNRNPK* | Hs00829140_s1 | 0.360 | 3 | 21 |
| *HNRNPM* | Hs00246018_m1 | 0.234 | 3 | 22 |
| *HNRNPUL2* | Hs00859848_m1 | 0.470 | 3 | 21 |
| *AKAP17A* | Hs00946624_m1 | 0.145 | 9 | 22 |
| *PNISR* | Hs00369090_m1 | 0.305 | 3 | 19 |
| *SRSF1* | Hs00199471_m1 | 0.191 | 3 | 20 |
| *SRSF2* | Hs00427515_g1 | 0.092 | 3 | 24 |
| *SRSF3* | Hs00751507_s1 | 0.195 | 3 | 22 |
| *SRSF6* | Hs00607200_g1 | 0.293 | 3 | 22 |
| *SRSF7* | Hs00196708_m1 | 0.217 | 3 | 20 |
| *TRA2B* | Hs00907493_m1 | 0.165 | 3 | 21 |
| *IMP3* | Hs00251000_s1 | 0.259 | 3 | 23 |
| *LSM14A* | Hs00385941_m1 | 0.146 | 3 | 21 |
| *LSM2* | Hs01061967_g1 | 0.191 | 3 | 23 |
| *SF3B1* | Hs00202782_m1 | 0.424 | 3 | 20 |
| ***18S*** | ***Hs99999901_s1*** | ***0.189*** | ***2*** | ***6*** |
| ***GUSB*** | ***Hs00939627_m1*** | ***0.249*** | ***8*** | ***22*** |
| ***IDH3B*** | ***Hs00199382_m1*** | ***0.249*** | ***3*** | ***22*** |
| ***PPIA*** | ***Hs04194521_s1*** | ***0.332*** | ***3*** | ***23*** |

**Supplementary Table S2: Associations of splicing factor expression with decline in corrected MMSE score as a continuous measure.**

Relationships of splicing factor expression levels with decline in corrected MMSE score by multivariate linear regression. β-coefficients represent change in log expression per unit change in MMSE score. Transcripts showing nominally statistically significant associations (*p*<0.05) are shown in italic and underlined. Those which satisfy Bonferroni correction for multiple testing (*p*<0.0083) are shown in bold italic and underlined.

| **DECLINE IN MMSE TEST SCORE (n = 296)** | | | | | | |
| --- | --- | --- | --- | --- | --- | --- |
|  |  | **β-coefficient** | **SE** | ***p*-value** | **95% CI Lower** | **95% CI upper** |
| **Splicing Factors** | *HNRNPA0* | *-0.003* | *0.001* | *0.019* | *-0.006* | *-0.001* |
|  | *HNRNPA1* | -0.001 | 0.002 | 0.476 | -0.005 | 0.002 |
|  | *HNRNPA2B1* | 0.000 | 0.001 | 0.783 | -0.003 | 0.002 |
|  | *HNRNPD* | 0.000 | 0.001 | 0.917 | -0.003 | 0.003 |
|  | *HNRNPH3* | -0.001 | 0.002 | 0.577 | -0.004 | 0.002 |
|  | *HNRNPK* | -0.001 | 0.002 | 0.488 | -0.005 | 0.002 |
|  | *HNRNPM* | ***-0.005*** | ***0.002*** | ***0.006*** | ***-0.008*** | ***-0.001*** |
|  | *HNRNPUL2* | -0.002 | 0.002 | 0.137 | -0.005 | 0.001 |
|  | *AKAP17A* | -0.005 | 0.003 | 0.077 | -0.011 | 0.001 |
|  | *PNISR* | 0.001 | 0.001 | 0.359 | -0.002 | 0.004 |
|  | *SRSF1* | -0.001 | 0.001 | 0.176 | -0.003 | 0.001 |
|  | *SRSF2* | -0.001 | 0.003 | 0.612 | -0.006 | 0.004 |
|  | *SRSF3* | 0.001 | 0.002 | 0.732 | -0.004 | 0.005 |
|  | *SRSF6* | -0.003 | 0.002 | 0.076 | -0.007 | 0.000 |
|  | *SRSF7* | 0.001 | 0.001 | 0.375 | -0.002 | 0.004 |
|  | *TRA2B* | 0.002 | 0.002 | 0.222 | -0.001 | 0.006 |
| **Core Spliceosome** | *IMP3* | 0.002 | 0.001 | 0.190 | -0.001 | 0.005 |
|  | *LSM14A* | 0.000 | 0.002 | 0.964 | -0.003 | 0.003 |
|  | *LSM2* | 0.004 | 0.002 | 0.111 | -0.001 | 0.008 |
|  | *SF3B1* | 0.001 | 0.002 | 0.717 | -0.003 | 0.004 |

**Supplementary Table S3: Further analysis of associations of splicing factor expression found with decline in MMSE score.**

Relationships of splicing factor expression levels with different subsets of the cohort by multivariate linear regression. β-coefficients represent change in log expression per unit change in MMSE score. Transcripts showing nominally statistically significant associations (*p*<0.05) are shown in italic and underlined. Those which satisfy Bonferroni correction for multiple testing (*p*<0.0083) are shown in bold italic and underlined.

| **SPLICING FACTORS ASSOCIATED WITH DECLINE IN MMSE SCORE** | | | | | | | |
| --- | --- | --- | --- | --- | --- | --- | --- |
| ***AKAP17A*** | | **n** | **β-coefficient** | **SE** | ***p*-value** | **95% CI Lower** | **95% CI upper** |
| Full cohort |  | 296 | -0.005 | 0.003 | 0.077 | -0.011 | 0.001 |
| FU3 MMSE score >=28 |  | 174 | -0.012 | 0.007 | 0.070 | -0.025 | 0.001 |
| FU3 Age >= 70 |  | 178 | -0.005 | 0.004 | 0.133 | -0.013 | 0.002 |
| Non-decliners removed |  | 260 | *-0.008* | *0.004* | *0.036* | *-0.015* | *-0.001* |
| Full cohort - categorised | Mild decline | 103 | -0.017 | 0.025 | 0.486 | -0.066 | 0.031 |
|  | Severe decline | 13 | ***-0.153*** | ***0.056*** | ***0.007*** | ***-0.264*** | ***-0.041*** |
| ***HNRNPA0*** | | **n** | **β-coefficient** | **SE** | ***p*-value** | **95% CI Lower** | **95% CI upper** |
| Full cohort |  | 296 | *-0.003* | *0.001* | *0.019* | *-0.006* | *-0.001* |
| FU3 MMSE score >=28 |  | 174 | *-0.007* | *0.003* | *0.010* | *-0.012* | *-0.002* |
| FU3 Age >= 70 |  | 178 | *-0.004* | *0.002* | *0.028* | *-0.007* | *0.000* |
| Non-decliners removed |  | 260 | ***-0.004*** | ***0.002*** | ***0.007*** | ***-0.008*** | ***-0.001*** |
| Full cohort - categorised | Mild decline | 103 | 0.004 | 0.011 | 0.702 | -0.018 | 0.026 |
|  | Severe decline | 13 | ***-0.083*** | ***0.025*** | ***0.001*** | ***-0.133*** | ***-0.033*** |
| ***HNRNPM*** | | **n** | **β-coefficient** | **SE** | ***p*-value** | **95% CI Lower** | **95% CI upper** |
| Full cohort |  | 296 | ***-0.005*** | ***0.002*** | ***0.006*** | ***-0.008*** | ***-0.001*** |
| FU3 MMSE score >=28 |  | 174 | *-0.009* | *0.003* | *0.011* | *-0.016* | *-0.002* |
| FU3 Age >= 70 |  | 178 | *-0.005* | *0.002* | *0.011* | *-0.009* | *-0.001* |
| Non-decliners removed |  | 260 | ***-0.006*** | ***0.002*** | ***0.005*** | ***-0.009*** | ***-0.002*** |
| Full cohort - categorised | Mild decline | 103 | -0.010 | 0.013 | 0.454 | -0.036 | 0.016 |
|  | Severe decline | 13 | ***-0.082*** | ***0.031*** | ***0.008*** | ***-0.143*** | ***-0.022*** |

**Supplementary Table S4: Correlations between splicing factor expression levels.**

Pearson correlations of relationships between expression levels of all splicing factors measured.

|  | HNRNPA0 | HNRNPA1 | HNRNPA2B1 | HNRNPD | HNRNPH3 | HNRNPK | HNRNPM | HNRNPUL2 | AKAP17A | PNISR | SRSF1 | SRSF2 | SRSF3 | SRSF6 | SRSF7 | TRA2B | IMP3 | LSM14A | LSM2 | SF3B1 |
| --- | --- | --- | --- | --- | --- | --- | --- | --- | --- | --- | --- | --- | --- | --- | --- | --- | --- | --- | --- | --- |
| HNRNPA0 | 1 |  |  |  |  |  |  |  |  |  |  |  |  |  |  |  |  |  |  |  |
| HNRNPA1 | 0.161 | 1 |  |  |  |  |  |  |  |  |  |  |  |  |  |  |  |  |  |  |
| HNRNPA2B1 | -0.228 | -0.026 | 1 |  |  |  |  |  |  |  |  |  |  |  |  |  |  |  |  |  |
| HNRNPD | -0.021 | -0.043 | 0.028 | 1 |  |  |  |  |  |  |  |  |  |  |  |  |  |  |  |  |
| HNRNPH3 | -0.214 | -0.096 | 0.281 | 0.189 | 1 |  |  |  |  |  |  |  |  |  |  |  |  |  |  |  |
| HNRNPK | -0.188 | -0.352 | 0.039 | 0.221 | 0.338 | 1 |  |  |  |  |  |  |  |  |  |  |  |  |  |  |
| HNRNPM | 0.282 | -0.049 | -0.012 | -0.031 | -0.292 | 0.028 | 1 |  |  |  |  |  |  |  |  |  |  |  |  |  |
| HNRNPUL2 | 0.226 | -0.034 | 0.062 | 0.393 | 0.366 | 0.201 | 0.143 | 1 |  |  |  |  |  |  |  |  |  |  |  |  |
| AKAP17A | 0.239 | -0.117 | 0.096 | -0.246 | -0.180 | 0.131 | 0.440 | -0.047 | 1 |  |  |  |  |  |  |  |  |  |  |  |
| PNISR | -0.330 | 0.045 | 0.027 | 0.074 | 0.419 | 0.126 | -0.317 | -0.023 | -0.270 | 1 |  |  |  |  |  |  |  |  |  |  |
| SRSF1 | 0.107 | 0.152 | -0.133 | -0.154 | 0.108 | -0.229 | -0.164 | 0.011 | -0.241 | 0.131 | 1 |  |  |  |  |  |  |  |  |  |
| SRSF2 | 0.080 | 0.005 | -0.189 | -0.134 | -0.353 | -0.017 | 0.243 | 0.009 | 0.179 | -0.188 | 0.071 | 1 |  |  |  |  |  |  |  |  |
| SRSF3 | -0.493 | -0.125 | 0.007 | -0.149 | -0.030 | 0.186 | -0.205 | -0.401 | -0.078 | 0.361 | -0.077 | -0.038 | 1 |  |  |  |  |  |  |  |
| SRSF6 | 0.126 | 0.024 | 0.143 | -0.159 | -0.224 | -0.216 | 0.005 | -0.143 | 0.076 | -0.308 | 0.045 | -0.025 | -0.269 | 1 |  |  |  |  |  |  |
| SRSF7 | -0.241 | 0.158 | -0.060 | -0.179 | -0.140 | -0.368 | -0.218 | -0.204 | -0.369 | 0.107 | 0.195 | 0.035 | 0.232 | -0.019 | 1 |  |  |  |  |  |
| TRA2B | -0.386 | 0.107 | 0.018 | -0.192 | -0.068 | -0.193 | -0.369 | -0.369 | -0.289 | 0.176 | 0.058 | -0.103 | 0.451 | -0.026 | 0.498 | 1 |  |  |  |  |
| IMP3 | -0.080 | 0.131 | -0.318 | -0.090 | -0.312 | -0.362 | -0.125 | -0.327 | -0.292 | -0.068 | 0.064 | 0.015 | 0.246 | 0.013 | 0.326 | 0.318 | 1 |  |  |  |
| LSM14A | 0.149 | -0.126 | 0.109 | -0.020 | 0.238 | -0.181 | -0.095 | 0.362 | -0.071 | -0.135 | 0.185 | -0.264 | -0.507 | 0.125 | -0.174 | -0.280 | -0.239 | 1 |  |  |
| LSM2 | -0.126 | 0.120 | -0.171 | -0.186 | -0.337 | -0.296 | -0.088 | -0.441 | 0.024 | -0.104 | -0.057 | -0.015 | -0.006 | 0.076 | 0.134 | 0.156 | 0.265 | -0.050 | 1 |  |
| SF3B1 | -0.344 | -0.195 | -0.049 | 0.014 | 0.476 | 0.260 | -0.441 | 0.009 | -0.421 | 0.505 | 0.229 | -0.257 | 0.426 | -0.287 | 0.158 | 0.302 | -0.114 | 0.036 | -0.258 | 1 |

**Supplementary Table S5: Associations of splicing factor expression with alternate measures of cognitive ability.**

Relationships between expression levels of splicing factors implicated in decline in MMSE score and results of the Trail Making Test A, Trail Making Test B and the Purdue Pegboard Test, by multivariate linear regression. For the Trail Making Tests, β-coefficients represent change in log expression per minute increased time taken to complete the tests. For the Purdue Pegboard Test, β-coefficients represent change in log expression per unit change in total number of pegs placed on the board. Transcripts showing nominally statistically significant associations (*p*<0.05) are shown in italic and underlined. Those which satisfy Bonferroni correction for multiple testing (*p*<0.0083) are shown in bold italic and underlined.

| **ASSOCIATIONS WITH INCREASE IN TIME TO COMPLETE TRAIL MAKING TEST A** | | | | | | | |
| --- | --- | --- | --- | --- | --- | --- | --- |
| ***AKAP17A*** | | **n** | **β-coefficient** | **SE** | ***p*-value** | **95% CI Lower** | **95% CI upper** |
| Full cohort |  | 268 | *0.057* | *0.022* | *0.009* | *0.014* | *0.099* |
| FU3 Lowest quintile removed |  | 224 | *0.067* | *0.031* | *0.035* | *0.005* | *0.128* |
| FU3 Age >= 70 |  | 151 | *0.070* | *0.027* | *0.010* | *0.017* | *0.124* |
| Non-decliners removed |  | 247 | ***0.074*** | ***0.025*** | ***0.004*** | ***0.024*** | ***0.123*** |
| Full cohort - categorised | Mild decline | 21 | 0.048 | 0.045 | 0.289 | -0.041 | 0.136 |
|  | Severe decline | 57 | -0.049 | 0.031 | 0.117 | -0.110 | 0.012 |
| ***HNRNPA0*** | | **n** | **β-coefficient** | **SE** | ***p*-value** | **95% CI Lower** | **95% CI upper** |
| Full cohort |  | 268 | ***0.028*** | ***0.009*** | ***0.004*** | ***0.009*** | ***0.046*** |
| FU3 Lowest quintile removed |  | 224 | *0.033* | *0.013* | *0.015* | *0.007* | *0.059* |
| FU3 Age >= 70 |  | 151 | *0.029* | *0.011* | *0.014* | *0.006* | *0.051* |
| Non-decliners removed |  | 247 | ***0.031*** | ***0.011*** | ***0.005*** | ***0.009*** | ***0.052*** |
| Full cohort - categorised | Mild decline | 21 | -0.013 | 0.020 | 0.506 | -0.053 | 0.026 |
|  | Severe decline | 57 | *-0.027* | *0.014* | *0.044* | *-0.054* | *-0.001* |
| ***HNRNPM*** | | **n** | **β-coefficient** | **SE** | ***p*-value** | **95% CI Lower** | **95% CI upper** |
| Full cohort |  | 268 | 0.012 | 0.011 | 0.299 | -0.010 | 0.033 |
| FU3 Lowest quintile removed |  | 224 | 0.020 | 0.016 | 0.214 | -0.012 | 0.051 |
| FU3 Age >= 70 |  | 151 | 0.021 | 0.012 | 0.092 | -0.003 | 0.046 |
| Non-decliners removed |  | 247 | 0.024 | 0.013 | 0.064 | -0.001 | 0.049 |
| Full cohort - categorised | Mild decline | 21 | 0.015 | 0.023 | 0.506 | -0.030 | 0.060 |
|  | Severe decline | 57 | -0.005 | 0.016 | 0.770 | -0.036 | 0.027 |

**Supplementary Table S5: Continued.**

| **ASSOCIATIONS WITH INCREASE IN TIME TO COMPLETE TRAIL MAKING TEST B** | | | | | | | |
| --- | --- | --- | --- | --- | --- | --- | --- |
| ***AKAP17A*** | | **n** | **β-coefficient** | **SE** | ***p*-value** | **95% CI Lower** | **95% CI upper** |
| Full cohort |  | 179 | 0.034 | 0.025 | 0.174 | -0.015 | 0.083 |
| FU3 Lowest quintile removed |  | 159 | 0.015 | 0.031 | 0.636 | -0.047 | 0.077 |
| FU3 Age > 70 |  | 67 | -0.007 | 0.042 | 0.863 | -0.097 | 0.082 |
| Non-decliners removed |  | 160 | 0.019 | 0.030 | 0.523 | -0.040 | 0.078 |
| Full cohort - categorised | Mild decline | 52 | 0.028 | 0.035 | 0.422 | -0.041 | 0.097 |
|  | Severe decline | 37 | -0.001 | 0.045 | 0.982 | -0.089 | 0.088 |
| ***HNRNPA0*** | | **n** | **β-coefficient** | **SE** | ***p*-value** | **95% CI Lower** | **95% CI upper** |
| Full cohort |  | 179 | 0.011 | 0.011 | 0.315 | -0.011 | 0.033 |
| FU3 Lowest quintile removed |  | 159 | 0.002 | 0.013 | 0.894 | -0.024 | 0.027 |
| FU3 Age > 70 |  | 67 | -0.013 | 0.018 | 0.463 | -0.051 | 0.024 |
| Non-decliners removed |  | 160 | 0.005 | 0.012 | 0.672 | -0.019 | 0.030 |
| Full cohort - categorised | Mild decline | 52 | 0.002 | 0.015 | 0.909 | -0.028 | 0.031 |
|  | Severe decline | 37 | -0.032 | 0.019 | 0.084 | -0.069 | 0.004 |
| ***HNRNPM*** | | **n** | **β-coefficient** | **SE** | ***p*-value** | **95% CI Lower** | **95% CI upper** |
| Full cohort |  | 179 | *0.026* | *0.012* | *0.036* | *0.002* | *0.051* |
| FU3 Lowest quintile removed |  | 159 | 0.021 | 0.015 | 0.182 | -0.010 | 0.051 |
| FU3 Age >= 70 |  | 67 | 0.010 | 0.019 | 0.629 | -0.032 | 0.051 |
| Non-decliners removed |  | 160 | 0.016 | 0.015 | 0.288 | -0.014 | 0.045 |
| Full cohort - categorised | Mild decline | 52 | 0.006 | 0.017 | 0.749 | -0.029 | 0.040 |
|  | Severe decline | 37 | *-0.051* | *0.022* | *0.022* | *-0.095* | *-0.007* |

| **ASSOCIATIONS WITH DECLINE IN TOTAL NUMBER OF PEGS PLACED IN PURDUE PEGBOARD TEST** | | | | | | | |
| --- | --- | --- | --- | --- | --- | --- | --- |
| ***AKAP17A*** | | **n** | **β-coefficient** | **SE** | ***p*-value** | **95% CI Lower** | **95% CI upper** |
| Full cohort |  | 257 | *-0.005* | *0.002* | *0.012* | *-0.008* | *-0.001* |
| FU3 Lowest quintile removed |  | 212 | -0.004 | 0.002 | 0.079 | -0.008 | 0.000 |
| FU3 Age > 70 |  | 141 | ***-0.009*** | ***0.003*** | ***0.002*** | ***-0.015*** | ***-0.004*** |
| Non-decliners removed |  | 204 | -0.005 | 0.003 | 0.060 | -0.010 | 0.000 |
| Full cohort - categorised | Mild decline | 87 | *-0.062* | *0.027* | *0.024* | *-0.116* | *-0.008* |
|  | Severe decline | 48 | *-0.094* | *0.035* | *0.008* | *-0.164* | *-0.024* |
| ***HNRNPA0*** | | **n** | **β-coefficient** | **SE** | ***p*-value** | **95% CI Lower** | **95% CI upper** |
| Full cohort |  | 257 | *-0.002* | *0.001* | *0.047* | *-0.003* | *0.000* |
| FU3 Lowest quintile removed |  | 212 | -0.001 | 0.001 | 0.101 | -0.003 | 0.000 |
| FU3 Age > 70 |  | 141 | ***-0.004*** | ***0.001*** | ***0.002*** | ***-0.006*** | ***-0.001*** |
| Non-decliners removed |  | 204 | ***-0.003*** | ***0.001*** | ***0.002*** | ***-0.005*** | ***-0.001*** |
| Full cohort - categorised | Mild decline | 87 | -0.004 | 0.012 | 0.751 | -0.027 | 0.020 |
|  | Severe decline | 48 | *-0.035* | *0.015* | *0.025* | *-0.065* | *-0.004* |
| ***HNRNPM*** | | **n** | **β-coefficient** | **SE** | ***p*-value** | **95% CI Lower** | **95% CI upper** |
| Full cohort |  | 257 | *-0.002* | *0.001* | *0.044* | *-0.004* | *0.000* |
| FU3 Lowest quintile removed |  | 212 | -0.002 | 0.001 | 0.073 | -0.004 | 0.000 |
| FU3 Age >= 70 |  | 141 | -0.002 | 0.001 | 0.081 | -0.005 | 0.000 |
| Non-decliners removed |  | 204 | -0.002 | 0.001 | 0.242 | -0.004 | 0.001 |
| Full cohort - categorised | Mild decline | 87 | -0.001 | 0.014 | 0.926 | -0.029 | 0.027 |
|  | Severe decline | 48 | -0.021 | 0.019 | 0.259 | -0.058 | 0.016 |

**Supplementary Table S6: Correlations between trajectories in phenotypic test scores.**

Pearson correlations of relationships between calculated change variables for the different cognitive and physical measures used in the current study. Part a. shows correlations seen between the cognitive measures and part b. the physical measures.

**a.**

|  | **MMSE: DECLINE IN TEST SCORE** | **TMT-A: INCREASE IN TIME TO COMPLETE** | **TMT-B: INCREASE IN TIME TO COMPLETE** | **PURDUE PEGBOARD: DECLINE IN TOTAL NUMBER OF PEGS PLACED** |
| --- | --- | --- | --- | --- |
| **MMSE: DECLINE IN TEST SCORE** | 1 |  |  |  |
| **TMT-A: INCREASE IN TIME TO COMPLETE** | -0.381 | 1 |  |  |
| **TMT-B: INCREASE IN TIME TO COMPLETE** | -0.188 | 0.322 | 1 |  |
| **PURDUE PEGBOARD: DECLINE IN TOTAL NUMBER OF PEGS PLACED** | 0.295 | -0.329 | -0.157 | 1 |

**b.**

|  | **MEAN HAND-GRIP STRENGTH: DECLINE IN EXERTED FORCE (kg)** | **EPESE SPPB: DECLINE IN COMPOSITE SCORE** | **400m FAST WALK: DECLINE IN CALCULATED SPEED (m/s)** |
| --- | --- | --- | --- |
| **MEAN HAND-GRIP STRENGTH: DECLINE IN EXERTED FORCE (kg)** | 1 |  |  |
| **EPESE SPPB: DECLINE IN COMPOSITE SCORE** | 0.217 | 1 |  |
| **400m FAST WALK: DECLINE IN CALCULATED SPEED (m/s)** | 0.265 | 0.357 | 1 |

**Supplementary Table S7: Associations of splicing factor expression with mean hand-grip strength as a continuous measure.**

Relationships of splicing factor expression levels with mean hand-grip strength by multivariate linear regression. β-coefficients represent change in log expression per Kg change in mean hand-grip strength. Transcripts showing nominally statistically significant associations (*p*<0.05) are shown in italic and underlined.

| **DECLINE IN MEAN HAND-GRIP STRENGTH (n = 285)** | | | | | | |
| --- | --- | --- | --- | --- | --- | --- |
|  |  | **β-coefficient** | **SE** | ***p*-value** | **95% CI Lower** | **95% CI upper** |
| **Splicing Factors** | *HNRNPA0* | 0.001 | 0.001 | 0.664 | -0.002 | 0.003 |
|  | *HNRNPA1* | 0.000 | 0.002 | 0.885 | -0.003 | 0.003 |
|  | *HNRNPA2B1* | -0.002 | 0.001 | 0.134 | -0.004 | 0.001 |
|  | *HNRNPD* | 0.002 | 0.001 | 0.198 | -0.001 | 0.004 |
|  | *HNRNPH3* | 0.001 | 0.001 | 0.667 | -0.002 | 0.003 |
|  | *HNRNPK* | 0.001 | 0.002 | 0.333 | -0.002 | 0.004 |
|  | *HNRNPM* | -0.002 | 0.001 | 0.256 | -0.004 | 0.001 |
|  | *HNRNPUL2* | 0.002 | 0.001 | 0.111 | 0.000 | 0.005 |
|  | *AKAP17A* | *-0.006* | *0.003* | *0.023* | *-0.011* | *-0.001* |
|  | *PNISR* | 0.000 | 0.001 | 0.707 | -0.002 | 0.003 |
|  | *SRSF1* | -0.001 | 0.001 | 0.480 | -0.002 | 0.001 |
|  | *SRSF2* | 0.001 | 0.002 | 0.771 | -0.004 | 0.005 |
|  | *SRSF3* | 0.000 | 0.002 | 0.932 | -0.004 | 0.004 |
|  | *SRSF6* | -0.001 | 0.002 | 0.469 | -0.004 | 0.002 |
|  | *SRSF7* | 0.001 | 0.001 | 0.547 | -0.002 | 0.003 |
|  | *TRA2B* | -0.002 | 0.002 | 0.278 | -0.005 | 0.001 |
| **Core Spliceosome** | *IMP3* | 0.001 | 0.001 | 0.672 | -0.002 | 0.003 |
|  | *LSM14A* | 0.000 | 0.001 | 0.759 | -0.002 | 0.003 |
|  | *LSM2* | 0.000 | 0.002 | 0.876 | -0.004 | 0.004 |
|  | *SF3B1* | 0.002 | 0.001 | 0.179 | -0.001 | 0.005 |

**Supplementary Table S8: Further analysis of associations of splicing factor expression found with decline in mean hand-grip strength.**

Relationships of splicing factor expression levels with different subsets of the cohort by multivariate linear regression. β-coefficients represent change in log expression per Kg change in mean hand-grip strength. Transcripts showing nominally statistically significant associations (*p*<0.05) are shown in italic and underlined. Those which satisfy Bonferroni correction for multiple testing (*p*<0.0083) are shown in bold italic and underlined.

| **ASSOCIATIONS WITH DECLINE IN MEAN HAND-GRIP STRENGTH** | | | | | | | |
| --- | --- | --- | --- | --- | --- | --- | --- |
| ***AKAP17A*** | | **n** | **β-coefficient** | **SE** | ***p*-value** | **95% CI Lower** | **95% CI upper** |
| Full cohort |  | 285 | *-0.006* | *0.003* | *0.023* | *-0.011* | *-0.001* |
| Mean FU3 hand-grip >= EWGSOP cutoffs |  | 204 | *-0.007* | *0.003* | *0.035* | *-0.013* | *0.000* |
| FU3 Age >= 70 |  | 169 | ***-0.011*** | ***0.004*** | ***0.008*** | ***-0.018*** | ***-0.003*** |
| Non-decliners removed |  | 229 | -0.005 | 0.004 | 0.190 | -0.012 | 0.002 |
| Full cohort - categorised | Mild decline | 74 | -0.026 | 0.029 | 0.370 | -0.084 | 0.031 |
|  | Severe decline | 52 | -0.053 | 0.031 | 0.094 | -0.115 | 0.009 |

**Supplementary Table S9: Associations of splicing factor expression with alternate measures of physical ability.**

Relationships between expression levels of splicing factors implicated in decline in mean hand-grip strength and physical performance as measured by the Epidemiologic Studies of the Elderly – Short Physical Performance Battery (EPESE SPPB) and a timed 400m fast walk, by multivariate linear regression. For the EPESE SPPB, β-coefficients represent change in log expression per unit decline in composite score. For the 400m fast walk, β-coefficients represent change in log expression per unit decline in speed calculated in m/s. Transcripts showing nominally statistically significant associations (*p*<0.05) are shown in italic and underlined. Those which satisfy Bonferroni correction for multiple testing (*p*<0.0083) are shown in bold italic and underlined.

| **ASSOCIATIONS WITH DECLINE IN EPESE SPPB COMPOSITE SCORE** | | | | | | | |
| --- | --- | --- | --- | --- | --- | --- | --- |
| ***AKAP17A*** | | **n** | **β-coefficient** | **SE** | ***p*-value** | **95% CI Lower** | **95% CI upper** |
| Full cohort |  | 276 | *-0.011* | *0.006* | *0.048* | *-0.022* | *0.000* |
| FU3 Lowest quintile removed |  | 216 | -0.002 | 0.009 | 0.810 | -0.020 | 0.016 |
| FU3 Age >= 70 |  | 160 | *-0.014* | *0.007* | *0.042* | *-0.027* | *-0.001* |
| Non-decliners removed |  | 244 | -0.012 | 0.006 | 0.068 | -0.024 | 0.001 |
| Full cohort - categorised | Mild decline | 61 | 0.028 | 0.031 | 0.356 | -0.032 | 0.089 |
|  | Severe decline | 46 | *-0.087* | *0.034* | *0.011* | *-0.155* | *-0.020* |

| **ASSOCIATIONS WITH DECLINE IN** **CALCULATED SPEED (m/s) DURING 400m FAST WALK** | | | | | | | |
| --- | --- | --- | --- | --- | --- | --- | --- |
| ***AKAP17A*** | | **n** | **β-coefficient** | **SE** | ***p*-value** | **95% CI Lower** | **95% CI upper** |
| Full cohort |  | 206 | *-0.252* | *0.101* | *0.013* | *-0.451* | *-0.053* |
| FU3 Lowest quintile removed |  | 183 | *-0.277* | *0.112* | *0.015* | *-0.499* | *-0.055* |
| FU3 Age >= 70 |  | 93 | -0.172 | 0.182 | 0.348 | -0.539 | 0.194 |
| Non-decliners removed |  | 194 | -0.189 | 0.117 | 0.109 | -0.421 | 0.043 |
| Full cohort - categorised | Mild decline | 126 | -0.053 | 0.034 | 0.118 | -0.120 | 0.014 |
|  | Severe decline | 39 | -0.084 | 0.046 | 0.068 | -0.174 | 0.006 |
